# Policy strategies for capacity building and scale up of the workforce for comprehensive cancer care: a systematic review

**DOI:** 10.1016/j.esmoop.2024.102946

**Published:** 2024-03-19

**Authors:** D. Trapani, S.S. Murthy, N. Hammad, R. Casolino, D.C. Moreira, F. Roitberg, J.-Y. Blay, G. Curigliano, A.M. Ilbawi

**Affiliations:** 1 Department of Oncology and Hemato-Oncology, University of Milano, Milano; 2 European Institute of Oncology, IRCCS, Milan, Italy; 3 Global Cancer Disparities Initiative, Division of Surgical Oncology, Memorial Sloan Kettering Cancer Center, New York, USA; 4 Michael’s Hospital, University of Toronto, Toronto, Canada; 5 Department of Noncommunicable Diseases, World Health Organization, Geneva, Switzerland; 6 Department of Global Pediatric Medicine, St. Jude Children’s Research Hospital, Memphis, USA; 7 Hospital Sírio-Libanês, São Paulo, Brazil; 8 Department of Medical Oncology, Centre Leon Berard, Lyon, France

**Keywords:** cancer workforce, capacity-building, global oncology, cancer policy, SWOT, AAAQ, WHO Strategy

## Abstract

**Background:**

Patients with cancer in low- and middle-income countries experience worse outcomes as a result of the limited capacity of health systems to deliver comprehensive cancer care. The health workforce is a key component of health systems; however, deep gaps exist in the availability and accessibility of cancer care providers.

**Materials and methods:**

We carried out a systematic review of the literature evaluating the strategies for capacity building of the cancer workforce. We studied how the policy strategies addressed the availability, accessibility, acceptability, and quality (AAAQ) of the workforce. We used a strategic planning framework (SWOT: strengths, weaknesses, opportunities, threats) to identify actionable areas of capacity building. We contextualized our findings based on the *WHO 2030 Global Strategy on Human Resources for Health*, evaluating how they can ultimately be framed in a labour market approach and inform strategies to improve the capacity of the workforce (PROSPERO: CRD42020109377).

**Results:**

The systematic review of the literature yielded 9617 records, and we selected 45 eligible papers for data extraction. The workforce interventions identified were delivered mostly in the African and American Regions, and in two-thirds of cases, in high-income countries. Many strategies have been shown to increase the number of competent oncology providers. Optimization of the existing workforce through role delegation and digital health interventions was reported as a short- to mid-term solution to optimize cancer care, through quality-oriented, efficiency-improving, and acceptability-enforcing workforce strategies. The increased workload alone was potentially detrimental. The literature on retaining the workforce and reducing brain drain or attrition in underserved areas was commonly limited.

**Conclusions:**

Workforce capacity building is not only a quantitative problem but can also be addressed through quality-oriented, organizational, and managerial solutions of human resources. The delivery of comprehensive, acceptable, and impact-oriented cancer care requires an available, accessible, and competent workforce for comprehensive cancer care. Efficiency-improving strategies may be instrumental for capacity building in resource-constrained settings.

## Highlights

• Shortages of the oncology workforce are common in low- and middle-income countries (LMICs).
• Strategies for capacity building of the cancer workforce must be evidence based and impact oriented.
• Most common strategies to improve capacity are educational and aim at increasing the number of providers.
• Organizational approaches, such as role delegation and digital health solutions, are key to improving the workforce capacity.
• More efforts are needed toward accountability and monitoring, and to enhance the retention of the workforce in LMICs.

## Introduction

Cancer is a leading cause of morbidity, mortality, and disability worldwide.^1^ The global cancer burden rose to 19.3 million new cases and 10 million deaths in 2020 and is predicted to rise to 28.4 million new cases by 2040. The majority of deaths are expected in low- and middle-income countries (LMICs),^1^ where patients experience poorer oncological outcomes, attributed to advanced disease presentation, and delays in timely access to safe, high-quality, and affordable health care,^2^ as a result of stark inequalities in health system capacities, resource constraints, and the availability and accessibility to a competent and resilient workforce. A skilled, multidisciplinary cancer workforce is vital to developing impact-oriented oncology programs.^3^ However, the cancer workforce is highly variable across the globe, with large differences in capacity to deliver comprehensive cancer care.^3^ The paucity of human resources is a common denominator of weaker health systems, ultimately affecting population health and cancer outcomes.^4^ By 2030, a shortfall of 18 million health workers has been projected, predominantly in LMICs, urging evidence-based, cost-effective strategies to build and scale up the capacity of the oncology workforce.^5^ In support of the policy formulation to tackle shortages of the cancer workforce, we carried out a systematic review of the literature to determine strategies used to build capacity and scale up the cancer workforce.

## Materials and methods

A systematic review of the literature was carried out, searching for publications on interventions that capture capacity building and scale up of the oncology workforce. The review was registered with PROSPERO (CRD42020109377). Two authors (DT and AMI) independently screened a set of six unique electronic databases (PubMed, Web of Science, SCOPUS, Embase, Google Scholar, and Cochrane Database of Systematic Reviews), with no restriction in language, for literature published between January 2008 and March 2023. Mapped MeSH terms were ‘workforce’, ‘health manpower’, ‘cancer’, and ‘oncology’. Grey literature was also evaluated, retrieved from online governmental reports, public health institutions, agencies, and other health policy documents. Relevant papers, not captured in our primary database search, were identified manually using a snowballing method.^6^ The selection process was carried out according to the Preferred Reporting Items for Systematic Review and Meta-Analysis (PRISMA) methodology.^7^ Mendeley (Mendeley Ltd., Elsevier, Amsterdam, The Netherlands) was used for title–abstract collection, management, and removal of duplicates. Manual screening of papers was carried out using the systematic review management software Rayyan (Qatar Foundation/Qatar Computing Research Institute, Ar-Rayyan, Qatar). The primary review authors (AMI and DT) independently screened the papers by titles and abstracts. Discordant determinations were adjudicated by a third independent reviewer (GC). All observational and/or interventional studies in public health or medicine describing the development, implementation, and scale up of interventions for the cancer workforce were considered for inclusion, regardless of the tumour types. Studies that were not specific to the cancer workforce were excluded, including articles that focused on cancer research development, descriptive policy reviews, and studies that lacked results on capacity-building outcomes. The search strategy was framed in accordance with the cancer continuum, extracting interventions from early detection to supportive and palliative care. For each study, the following information was extracted: country and year of strategy implementation, type of sponsorship and funding, speciality of providers, study design and methodology of data collection, and the health care setting. Countries were grouped by World Health Organization (WHO) regions [American (AMRO), African (AFRO), European (EURO), Eastern-Mediterranean (EMRO), South-East Asian (SEARO), and West-Pacific (WPRO)] and World Bank (WB) income groups [low- (LIC), LMIC, upper-middle- (UMIC), and high- (HIC) income country] (Supplementary Table S1, available at https://doi.org/10.1016/j.esmoop.2024.102946).

The relevant information was extracted in an Excel spreadsheet and analysed using the AAAQ framework to understand the impact of the interventions on Availability, Accessibility, Acceptability, and Quality of the workforce.^8^ The strengths, weaknesses, opportunities, and threats (SWOT) matrix was completed to evaluate the strengths, weaknesses, opportunities, and threats of the interventions.^9^^,^^10^ We contextualized the workforce interventions within the *WHO 2030 Global Strategy on Human Resources for Health*,^11^ the WHO policy guidance for development and scale up of the workforce, based on a labour market approach (Supplementary Figure S1, available at https://doi.org/10.1016/j.esmoop.2024.102946). Eventually, to understand the impact of single workforce policy strategies, we evaluated how these policies would contribute to the development of five standardized modelling scenarios, an approach previously used by the WHO to guide policymakers on actions that can build workforce capacity.^12^ Scenarios describe policies capable of (i) increasing the number of skilled providers, (ii) reducing the voluntary attrition rate, (iii) addressing increasing new cancer diagnosis, (iv) increasing intervention efficiency, and (v) optimizing the existing workforce.^11^ Descriptive statistics were used to summarize study characteristics.

## Results

### Overview

**Figure 1:**
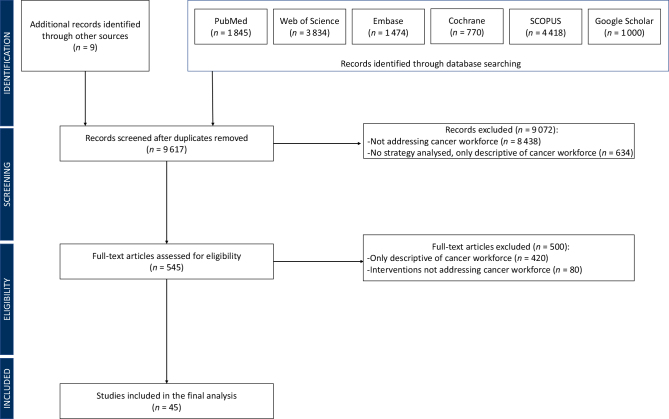
Preferred Reporting Items for Systematic Review and Meta-Analysis (PRISMA) flow chart of the systematic review.

**Figure 2:**
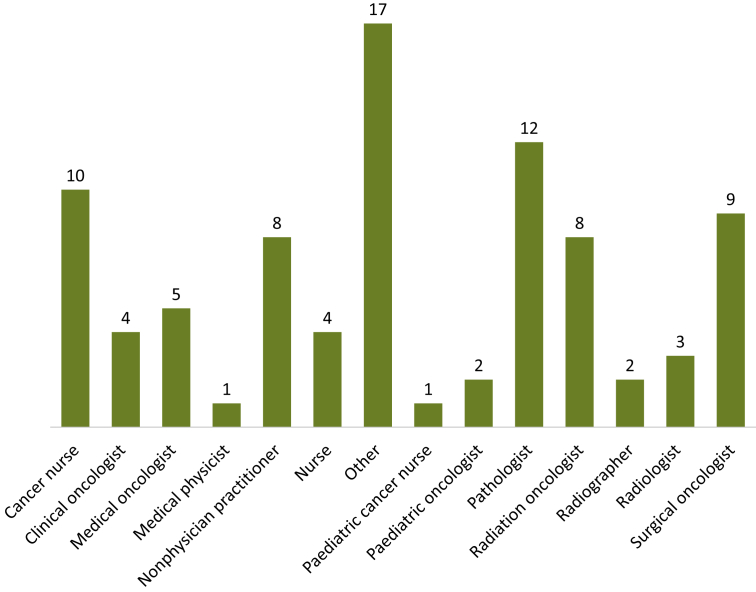
**Health providers addressed by the workforce interventions in the systematic review.** Note: A single paper could report a strategy intended for a single or multiple types of providers. Surgical oncologists include one neurosurgeon. Other: multidisciplinary team (occupations not specified), native health workers, health care professionals in supportive care of patients with cancer, medical social worker, one data manager, one outreach worker, local medical officer, assistant medical officer, caregiver, ophthalmology medical officer, office staff, social worker, community health advisors, rural health care providers, patient navigator, primary care physician, medical student, and radiology nurse.

**Figure 3:**
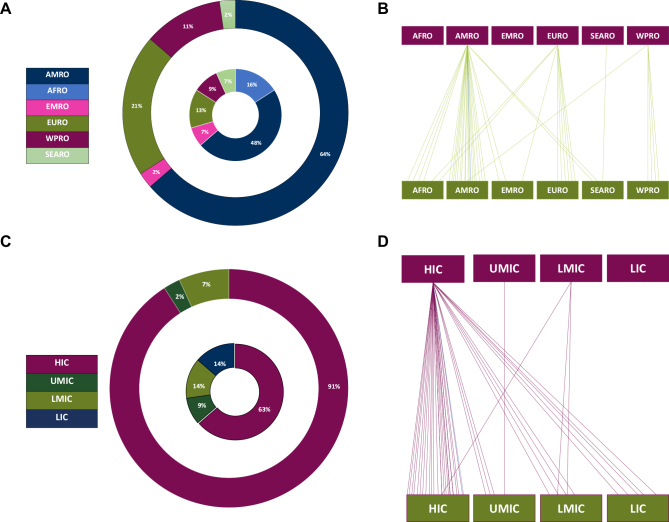
**Countries and institutions where the workforce strategies were formulated, funded, and implemented**. (A) Distribution of the countries where the workforce strategies were formulated (outer circle) and implemented (inner circle), according to the WHO Region. (B) Countries where the funding institutions were based (above) and countries where the workforce strategies were implemented (below), according to the WHO Region. (C) Distribution of the countries where the workforce strategies were formulated (outer circle) and implemented (inner circle), according to the WB income grouping. (D) Countries where the funding institutions were based (above) and countries where the workforce strategies were implemented (below), according to the WB income groupings. WHO Regions: AFRO, African; AMRO, American; EMRO, Eastern-Mediterranean; EURO, European; SEARO, South-East Asian; WPRO, West-Pacific. WB grouping: HIC, high-income country; LIC, low-income country; LMIC, lower-middle income country; UMIC, upper-middle income country. WB, World Bank; WHO, World Health Organization.

We screened 9617 unique records and 45 studies ultimately met the inclusion criteria (PRISMA flow chart in Figure 1). Two-thirds of the studies (*n* = 30) reported strategies to build and scale-up capacity for a single type of health provider, including physicians (*n* = 16) and nonphysicians (*n* = 14) (Figure 2; Supplementary Figure S2, available at https://doi.org/10.1016/j.esmoop.2024.102946). Countries where workforce interventions were implemented were most commonly in the WHO Region of the Americas (AMRO; *n* = 21) and the African Region (AFRO; *n* = 7). HICs were more represented (*n* = 28, 63%) than LICs and LMICs (Figure 3B and D). Data on financial support for workforce interventions were mostly derived from HICs (*n* = 40) and the AMRO region (*n* = 28; Figure 3A and C). Countries tended to support other countries in the same WHO Region (70% of the cases) and in the same WB income group (67%; Figure 3A–D). The funding source was identified in 60% of the papers (*n* = 27): academic institutions were the most common financing entity (*n* = 21) (Supplementary Figure S3, available at https://doi.org/10.1016/j.esmoop.2024.102946). Programs implemented in LICs were exclusively designed by stakeholders from HICs (*n* = 5), largely from the AMRO region (*n* = 4; Figure 3C and D).

We found that interventions addressed both rural and urban health care settings, with 16% (*n* = 7 papers) of the studies intended for both settings. The delivery of health care intervention services was mainly carried out on-site (*n* = 27); however, 31% (*n* = 14) were designed as digital health interventions (Supplementary Figure S4, available at https://doi.org/10.1016/j.esmoop.2024.102946).

**Figure 4:**
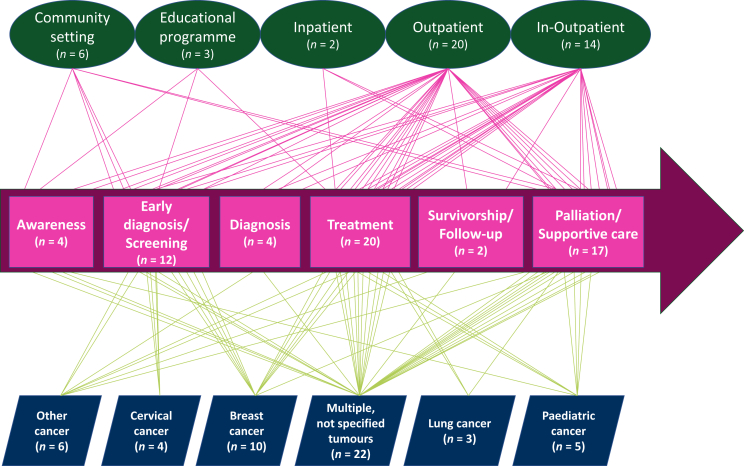
Workforce strategies identified in the systematic review across the cancer continuum of care.

In the context of the cancer continuum, two-thirds (*n* = 15) of the interventions were designed to strengthen the workforce in relation to cancer treatment and/or supportive and palliative care. Policy interventions to increase community awareness and survivorship care only represented a minority of the studies (*n* = 4). In 56% of the studies (*n* = 25), interventions aimed to strengthen the capacity for a specific cancer type, where breast cancer (36%), cervical cancer (15%), and paediatric cancers (18%) were the most represented. Interventions intended to scale-up the capacity of the workforce to deliver care for single cancers were mostly focused on screening and treatment; by contrast, workforce interventions for supportive care were not generally tumour specific (Figure 4).

A variety of methodological approaches to measure the impact of the health interventions were identified. The most common study design was the pre–post experimental design without randomization (*n* = 28; 62%); randomized controlled studies were a minority (*n* = 4)^13^, ^14^, ^15^, ^16^ (Supplementary Figure S5, available at https://doi.org/10.1016/j.esmoop.2024.102946). In-person interviews or questionnaires administered to the health personnel (*n* = 17) and a review of the medical records (*n* = 13) were used to assess outcomes.

### Availability–accessibility–acceptability–quality (AAAQ)

#### Availability

Lack of sufficient human resources was the primary driver in designing workforce interventions, namely, strategies pursuing availability, commonly aimed at increasing the number of skilled health providers through more training programs. Digital health platforms for education and training were common approaches used to deliver educational programs. A subset of the projects envisioned a rapid scale up of the workforce via managerial solutions, not intended to primarily increase the number of providers. Task shifting, task sharing, and role delegation were the most common organizational solutions identified, aiming at delegating competencies traditionally assigned to physicians to mid-level health providers (*n* = 10). These strategies were viewed as more efficient because they can deliver prompter solutions to the workforce shortage, and efficiently fill supply–demand gaps. Nurses and nurse practitioners were the object of interest for interventions based on role delegation in 70% of these studies (*n* = 7). The projects based on role delegation were all educational in nature, using training modules for strengthening selected competencies. Examples of proposed task switching were reported for cancer genetic counselling, breast clinical examination, referral of a patient with prostate cancer from primary health care, cancer follow-up, palliative care, and selected operational procedures such as cervical pap smear and mammography. In one study, task shifting to paediatric cancer nurses resulted in improved cancer survival for patients^17^ (Table 1).

**Table 1:** Synopsis of the studies extracted from the systematic review.

Occupations addressed	Cancer type	Major findings	Reference
Multidisciplinary team (occupations not specified)	Lung cancer	Lung cancer tele-multidisciplinary tumour board: the majority of participants (60%) thought the day and time of the virtual tumour board were convenient; 40% preferred virtual to face-to-face tumour boards.	Stevenson et al.^50^
Medical oncologist, surgical oncologist, pathologist cancer nurse, and radiation oncologist	Breast cancer, lung cancer, and genitourinary cancer	Participants agreed that the virtual tumour boards provided new information for improving care for patients with cancer (96%), with educational achievement (86%) through a major familiarity with evidence-based data from clinical trials (93%).	Bold et al.^20^
Cancer nurse and assistant medical officer	Cervical cancer	The Kilimanjaro method [smartphone-based cervicography and text message (image transfer) platform] to enhance visual inspection with acetic acid training and procedural quality and accuracy through real-time tele-mentorship. Role delegation (nurses and medical officers): response from experts was provided within 10 min, with 96.8% agreement between trainees and experts after 1 month of training.	Yeates et al.^51^
Urology nurse practitioner	Prostate cancer	Role delegation of nurse practitioners involved in a nurse-led clinic for suspected prostate cancer referrals: 87% of management was appropriate, 52% less waiting times, cost-reduction was demonstrated, and 95% of patients were satisfied.	Drudge-Coates et al.^52^
Nonmedical genetic counsellor	No	Role delegation of nonphysician genetic counsellors contributed to 95% of total patient contacts, providing 93.7% of initial contacts, with one-quarter of patients discharged at that point.	Benjamin et al.^53^
Clinical oncologist, cancer nurse, and radiologist	Breast cancer	The mobile mammography unit of Nimra provided screening for 646 patients, of whom 106 were suspicious of malignancy, 97 were benign, and 443 were normal.	Laghari et al.^54^
Medical oncologist	No	Medical tele-oncology project in the Townsville Cancer Centre provided cancer care to 70 new patients; 93% were seen within 1 week of referral.	Sabesan et al.^55^
NPPs	No	Role delegation of NPPs with different models: • Incident-to-practice model: NPPs routinely see patients independent of the physician; • Shared practice model: NPPs always see patients in conjunction with the physician. • Independent practice model: patients are assigned to the NPP and not assigned to an oncologist. 83% of the NPPs considered the workload to be appropriate; 98% of patients were satisfied.	Towle et al.^33^
Pathologist, intended as a geneticist	No	Total costs to provide genetic consultation were US$106.19 per telegenetics patient and US$244.33 per in-person patient; no difference in the satisfaction of patients.	Buchanan et al.^13^
Rural health care providers (not specified)	No	Satisfaction with telehealth was high (3.6 on a 4-point scale); the most well-attended sessions were psychological issues in American Indians and Alaska natives with cancer (*n* = 36), lymphomas (*n* = 20), and pain and symptom management in cancer (*n* = 20). Usefulness rated as 3.6.	Doorenbos et al.^56^
Cancer nurse, medical social worker, data manager, and outreach worker	Paediatric cancers	The introduction of new health personnel in an insufficient workforce setting in India, provided by an NGO, resulted in a better outcome for children with cancer: reduced abandonment of 37% and improved overall survival of patients, +32% absolute gain in overall survival.	Mehta et al.^57^
Nurse and local medical officer	No	Health workers described the benefits of teleoncology not only for patients and their families but also for adding educational value for themselves, with closer working relationships with the specialist team.	Mooi et al.^25^
Breast surgery nurse practitioners	Breast cancer	Role delegation to nursing practitioners of nonsurgical competencies of breast surgeons. Surgical procedures increased, reflecting an increase in surgical consults, while nursing practitioners were handling nonsurgical consults.	Kanumuri et al.^58^
Paediatric cancer nurse	Paediatric cancers	The nursing program for improving quality standards related to nursing education and staffing permitted the fulfilment of the Joint Commission International Standards from 5% to 80%; new nursing positions were created, increasing the nurse-to-patient ratio.	Day et al.^30^
Pathologist	No	The Komfo Anokye Teaching Hospital in Kumasi and University Hospital of North Norway training program to re-establishing a Surgical Pathology Service in Kumas: in 2008, two Ghanaian doctors were at the end of their second year of training for the pathology speciality; by the end of October 2007, 303 cases of cancer had been reported.	Stalsberg et al.^59^
Surgical oncologists and cancer and non-cancer surgeons	No	Surgical training partnership of the University of Guyana and the Canadian Association of General Surgery. All five residents successfully completed 2 years of training and passed the final examinations.	Cameron et al.^60^
Paediatric oncologist, neuro-radiologist, neurosurgeon, and radiation oncologist	Paediatric tumours (primary central nervous system)	The King Hussein Cancer Center in Jordan, in collaboration with a Canadian telemedicine partnership, established a teleoncology service for paediatric neuro-oncology cases. Recommendations for major changes from the original treatment plan were made in 36% of cases, with 91% of these recommendations being followed. This underscores the feasibility and flexibility of the service in facilitating appropriate clinical decisions, potentially impacting patient outcomes.	Qaddoumi et al.^61^
Clinical oncologist, medical oncologist, surgical oncologist, pathologist, cancer nurse, and radiation oncologist	Breast cancer	Cluster randomization-based study of telemedicine versus standard ‘in-person’ meetings. Levels of agreement among members in both the telemedicine and standard meetings for decision sharing, consensus, and confidence in the decision were high and similar.	Kunkler et al.^14^
Patient navigator	Breast cancer and gynaecologic tumours	Treatment adherence across randomized groups of written resource navigation information versus written information plus patient navigation suggested that active telephone patient navigation or written resource informational materials may facilitate adherence among low-income female patients with cancer.	Ell et al.^15^
Pathologist	No	The Bugando Medical Centre and the NGO *Pathologists Beyond Borders* worked for the establishment of an autonomous surgical pathology laboratory. A quality assessment revealed very high concordance for adult general pathological diagnoses (90%) and paediatric/adolescent pathological diagnoses (91.18%) with <6% of major discordances.	Tumino et al.^28^
General and cancer surgeon	No	The establishment of surgical services in rural areas of developing countries by using simple facilities, providing them with basic equipment, and using local personnel selected and trained on the job by teams comprising a consultant surgeon, anaesthetist, and scrub nurse if feasible. The ‘surgical missions’ presented an effective strategy to improve the surgery workforce for low-income rural areas, while reducing the ‘brain drain’ and attrition rate.	Meo et al.^16^
Radiology technician and radiology nurse	Breast cancer	On-site mobile mammography in addition to health education for older and lower-income women in the United States. Workforce estimated: 1 FTE radiographic technologist, one-third FTE receptionist, and 1/3 FTE scheduler. The stationary MammoRx unit is expected to perform twice as efficiently as the mobile MammoRx unit.	Naeim et al.^18^
Nurse practitioner	Bladder cancer	A tele-cystoscopy model in which nurse practitioners carry out cystoscopies (role delegation), which were interpreted and directed in real-time by board-certified urologists at a remote location; universal satisfaction of the patients. Minimal training estimated: 30 cystoscopies.	Lee et al.^62^
Pathologist	Cervical cancer	Pap smear cytology taught by online courses to medical students: There is a 75% concordance rate with the cytopathologist.	Dewar et al.^63^
Cancer nurse	No	The experience has yielded an educated and skilled oncology workforce at Butaro Hospital and has developed the first Rwandan oncology nurse leaders, a ward manager, a care coordinator, and an educator. Improvement of average knowledge in oncology subjects.	Muhayimana et al.^26^
Community health advisors	No	Role delegation to develop a curriculum for community health advisors focusing on key competencies for lay navigation in the principles of palliative care has resulted in significant improvements. Over 50% of participants showed enhancements in their ability to discuss advanced illness, their self-efficacy, and their initiation of goals of care discussions.	Kvale et al.^64^
Radiation oncologist and radiation therapist	No	Remote radiation therapy treatment planning led to a significant decrease in turnaround time and reduced the need for on-call support, thereby enhancing clinical workflow and efficiency. The ability to work remotely from home promotes workforce flexibility and helps retain professionals in the field.	Enge et al.^21^
Health care professionals in supportive care	No	An online training program for palliative care was successful in improving the use of supportive care	Brady et al.^65^
Surgical oncologist	No	The Kamuzu Central Hospital Surgery Residency program began in 2009 with three residents, adding three general surgery and two orthopaedic residents in 2010. Educational training directly provided to surgery residents, along with support for local staff surgeons, was implemented in tandem with monetary assistance.	Qureshi et al.^66^
Pathologist	Breast cancer and melanoma	The financial potential of cost reduction through digital pathology was evaluated for melanoma and breast cancer diagnosis. An estimated 13% increase in productivity, coupled with a 50% reduction in annual internal secondary consults, resulted in cost savings of US$5.35 million. This was attributed to improved diagnoses and the avoidance of unnecessary treatments.	Ho et al.^67^
Radiation oncologists, medical physicists, and radiation therapists	No	A comprehensive capital investment strategy coupled with increased investments in human resource planning resulted in an increase in patients treated (>38%).	Ang et al.^68^
Radiographer (radiologic technologist)	Breast cancer	The role delegation of radiographers to interpret screening mammography, following a 6-month training period in a screening setting, led to similar diagnostic sensitivity compared with US radiologists, but higher false-positive rates were observed. A scenario in which a radiographer reads all mammograms first, and a radiologist reads only those that were difficult for the radiographer, was more cost-effective than a scenario in which either the radiographer or radiologist reads all mammograms.	Torres-Mejía et al.^69^
Clinical oncologist, medical oncologist, surgical oncologist, pathologist, paediatric oncologist, radiation oncologist, ophthalmic clinical officers, and nurses	Retinoblastoma	Interactive Workshop for Clinicians in Kenya on cancer genetics. Participant comments indicated that they found the lecture material and the role-play useful and relevant to their practices. Respondents indicated that they found the material informative and appreciated the testimonials of affected families.	Hill et al.^70^
Pathologist	Cervical cancer	The fully automated computer-assisted Pap test is an accepted and reliable cytology machine-assisted screening method for cervical cancer; when screening volume is >49 000 slides/year, the cytologist productivity increases about threefold, reducing human costs.	Della Palma et al.^71^
Cancer nurse	No	In 2011 alone, the 168 nurses trained under the Cancer Prevention and Research Institute of Texas grant reported that they educated or carried out clinical breast examinations on >7500 women (role delegation). An additional 424 clinicians were trained during in-house modules, resulting in further education of patients and the performance of procedures such as breast examinations, Papanicolaou tests, and colposcopies.	Dallred et al.^72^
Pathologist	Yes, cervical cancer	The increase in cytotechnologist workload above 100 slides per day using the ThinPrep resulted in worse diagnostic performance.	Elsheikh et al.^73^
Primary care physicians, nurses, social workers, and office staff	No	A competency-based approach to expanding the palliative care workforce through multidisciplinary and video-assisted educational programs resulted in +21% increase in the overall level of confidence: 90% reported improvement in gaining new knowledge and skills to provide better palliative care.	Cox et al.^24^
Medical students	Paediatric tumours	A competency-based approach to expanding the paediatric supportive care workforce through multidisciplinary and video-assisted educational programs resulted in +28% increase in knowledge, 331% increase in confidence in assessing pain in paediatric patients, 403% increase in confidence in treating pain in paediatric patients, and 255% increase in confidence in the ability to prescribe opioids to treat pain in paediatric patients.	Cox et al.^24^
Medical assistants and nurses	No	Nurses and medical assistants practising in rural, long-term care facilities; +12% increase in knowledge from pre- to post-test scores.	Cox et al.^24^
Native health workers and caregivers (from the five tribes)	No	Culture-Specific Pain Management program resulted in +120% improvement in confidence to identify and report symptoms in tribal settings.	Cox et al.^24^
Pathologist	Breast cancer	At the University of Chicago and the Institute for Advanced Medical Research and Training in Ibadan, immunohistochemistry was conducted on breast specimens. Initially, a moderate to fair concordance was observed for hormone receptor and HER2 expression in breast cancer samples. However, following educational training via a web-designed platform for pathology interpretation, the agreement improved substantially, with Cohen κ coefficient scores increasing from 0.39-0.42 to 0.6-0.75.	Oluwasola et al.^74^
Cancer nurse	No	Global project of paediatric oncology nurses education and clinical training resulting in higher retention rate.	Wilimas et al.^75^
Cancer nurse	Lung cancer and mesothelioma	Nurses-led follow-up (role delegation) versus conventional medical follow-up in the management of patients with lung cancer; estimates of median survival time were similar: 9.2 versus 10.4 months (*P* = 0.99). Costs were not significantly increased (*P* = 0.66).	Moore and Sherwin^76^
Palliative care provider	No	Mobile phones for better management of patients with terminal cancer in rural Bengal: 76% of the cases were managed by phone, with only 24% of patients attending the nodal centre for palliative expert consultation.	Manna^22^

FTE, full-time equivalent; HER2, human epidermal growth factor receptor 2; NGO, nongovernmental organization; NPP, nonphysician practitioner.

#### Accessibility

The lack of an accessible workforce has been identified as one major barrier to delivering efficient health care.^3^ A number of workforce interventions were designed to improve capacity in rural, remote, and underserved areas. Clinical discussions such as virtual tumour boards and mobile health clinical units were the principal interventions identified to enhance accessibility. One study evaluated the performance of mobile mammography units in rural areas, describing improved access to imaging; however, it yielded a lower sensitivity and specificity, when compared with the clinic-based mammography in urban sites.^18^ Three studies reported experiences with virtual tumour boards for cancer management, discussing patients managed in underserved areas, with providers in tertiary centres.^19^, ^20^, ^21^ The online multidisciplinary discussion resulted in more consistent, guideline-adherent treatment decisions for patient care, improved education of the health care personnel, and patients’ satisfaction.^19^, ^20^, ^21^ In one study, the implementation of a mobile phone-based service for terminally ill patients with cancer residing in rural areas reduced inappropriate emergency visits to the hospital for symptom control in up to 76% of patients^22^ (Table 1).

#### Acceptability

Cultural understanding is integral in developing a cancer care workforce that is patient centred.^3^ An acceptable workforce delivers empowering health services and is aware of the demographic, social, cultural, and economic aspects of communities. A culturally sensitive approach, with educational materials adapted to a language that patients can understand, has demonstrated objective improvements in patients’ symptom reporting and efficacy of service delivery.^23^ Key studies on acceptability retrieved from our research focused on improving the workforce with regard to indigenous populations.^24^^,^^25^ Strategies based on an acceptability goal were intended to strengthen medical awareness and literacy, as a means to enhance access to cancer care for more vulnerable populations (Table 1).

#### Quality

Reinforcement of health competencies to produce a skilled cancer workforce was the common denominator of strategies with quality improvement goals. One study described an intervention via an on-site training program, which resulted in an objective increase in medical knowledge of the health personnel, as demonstrated with knowledge assessment tests before versus after interventions.^26^ Quality of the training was identified as the most important element in the design of strategies for task shifting/task sharing.^27^^,^^28^ Assurance of quality training programs based on international standardized metrics and indicators was identified as a key strategy to reduce the ‘brain drain’ phenomenon in rural or low-income settings. Interventions to guarantee high-quality, equal access to medical education were short-term exchanges or twinning programs, hybrid on-site, or remote training sessions for providers from institutions in underserved areas. A common goal for all interventions was to enhance workforce retention and tackle the attrition rate.^29^ A training program for nurses resulted in improved accreditation quality scores in a cancer facility in a UMIC, emphasizing the importance of providing quality training to the workforce which in turn affects patient outcomes.^30^ Conversely, another study showed that an increase in workload alone as a way to improve workforce capacity with disregard to the quality of the work as well as satisfaction of the providers led to poorer diagnostic performance.^22^ The strategy was based on an increase in the daily workload of laboratory cytotechnologists.^22^ Increasing the workload alone was deemed not an efficient evidence-based intervention for workforce capacity building, and misalignment with quality goals and empowerment of the health personnel appeared potentially detrimental to providers and their performance and, as a consequence, on the health care delivery (Table 1).

### Strengths–weaknesses–opportunities–threats (SWOT)

A shortage and maldistribution of the existing workforce was a common weakness identified in our analysis. Inefficient and lack of systematic organization of health care services were drivers of a weak workforce. Strategies addressed inefficiencies through structural interventions to fix health system weaknesses (*n* = 17 studies), mostly by training new personnel, or potentiating existing elements of strength (*n* = 9), by improving efficiency. The principal threats to implementation and to building capacity included context and cultural inappropriateness, in addition to scale-up mechanisms that did not involve measurement of quality metrics, and only aimed at increasing the numbers of the providers or their workload^22^ (Table 1).

### The labour market-based analysis contextual to the WHO 2030 Strategy

The labour market approach outlined in the *WHO 2030 Strategy* identifies critical actionable phases to increase the capacity of the workforce: production of health personnel, inflows and outflows management, maldistribution and inefficiencies, and regulation of the private sector. All the strategies analysed in our systematic research aimed and/or resulted in a change in the number of skilled providers and/or of the skill-mix composition. Positive outcomes derived from increased production of the workforce and managerial solutions, including through role delegation (Supplementary Figure S6, available at https://doi.org/10.1016/j.esmoop.2024.102946). A multitude of workforce interventions were intended to enhance skills in the use of medical technologies. Telehealth applications and mobile clinical and diagnostic units were commonly included in the workforce strategies, resulting in optimization of the workload distribution and of the competencies. Fewer studies were designed to reduce the voluntary attrition rates and control the emigration abroad of health personnel. These strategies were based on quality educational projects and continuous medical education. The empowerment of the health providers was identified as a key intervention to develop a satisfied and resilient workforce. We could not identify durable and strong examples of workforce retention packages; therefore, the long-term impact of these strategies appears less clear (Table 1).

## Discussion

We presented the results of a systematic review of the literature on strategies that have been implemented to build and scale-up cancer workforce capacity in recent years. To our knowledge, this is the first systematic review specifically evaluating the oncology workforce. We selected studies reporting outcomes that inform the formulation of health policy quantitatively and qualitatively. After a broad screening of multiple databases, we found limited evidence and interventions on how to build up the cancer care workforce. Most interventions have been implemented in AMRO and AFRO, and the majority are based on academic initiatives. Durability and sustainability of the interventions were not commonly reported, so it is unclear to what extent the projects discussed were time limited, and how their impact persisted beyond being case studies. We report that quality improvement and efficiency-oriented workforce investments can catalyse progress to face health demands related to the growing cancer burden. Efficiency improvement can be pursued with role-delegation approaches, in areas where the delivery of health care can be assured at high-quality standards, albeit delivered by a diverse workforce. To be functional in the community, health personnel should tailor cultural and socioeconomic population needs, by enforcing acceptability. An ultimate, durable, and long-term sustainable expansion of the workforce is needed in many settings. Importantly, short-term solutions based only on an increase in the workload appeared to be detrimental to the quality of the health services and, as a result, of the health outcomes. Our analysis was not able to identify robust solutions to tackle brain drain or reduce attrition rates, based on workforce retention packages. We emphasize the importance of developing a workforce registry, to enhance accountability, and to better understand distribution of the health providers. An important proportion of maldistribution is due to the internal brain drain of providers into the private sector, becoming pervasive and threatening to universal health care, equitable distribution of the workforce, and research productivity. In our research, it emerged that assuring equitable career opportunities and incentives for providers in underserved and rural areas may result in professional satisfaction, and probably in workforce retention.

Our analysis highlights the common misconception and simplistic view that the problem of insufficient workforce in LICs and MICs is only a quantitative problem, demanding quantitative solutions. The inadequate number of providers and the need for developing new personnel are indeed crucial aspects. However, they do not solely determine the potential impact on population health through workforce strategies. According to the WHO, actions for the workforce should be framed with a comprehensive intersectoral policy approach, from a labour market economic perspective.^11^ We extracted numerous articles with evidence demonstrating that investments aimed at improving the competency of the existing workforce can expand the scope of the workforce through quality-oriented training programs for mid-level providers and offering organizational solutions that might efficiently optimize workforce capacity and distribution.^31^ In particular, role delegation has demonstrated the potential to provide a short- to mid-term solution to optimize the skill mix. The implementation of role delegation has appeared feasible, acceptable, and able to portend population health benefits in noncancer settings. For example, a program of nurse-led services to deliver antiretroviral therapy for human immunodeficiency virus (HIV) showed noninferior patients’ adherence to treatments and viral suppression; similarly, for the management of hypertension in the primary health care setting, mid-level practitioners delivered a patient care with similar outcomes as for physician-led care.^32^ However, data on the long-term impact are commonly lacking.^33^^,^^34^ Assuring competence-driven quality health services is instrumental in delivering impactful health care with better clinical outcomes. When quality is not central in capacity building and scale-up strategies, the health service performance is eroded, with frustration and disengagement of the health care personnel that can lead to poor outcomes for patients. Poor-quality services cause up to 60% of global excess deaths amenable to health care, representing a key determinant of population health.^35^

Our research also emphasizes the importance of acceptability in programming the cancer workforce. Addressing social and economic determinants of health is a priority for policy involving workforce development and capacity building. A lack of an acceptable workforce means a lack of inclusivity, leading to health care exclusion and hesitation to seek help, for those in need. Of note, we could not identify specific interventions aiming at improving acceptability for gender minorities, urging more solutions toward all minorities.^36^

We found very limited evidence on strategies designed to reduce migration and voluntary attrition.^37^^,^^38^ We believe that any policy should shape retention packages based on economic, social, and educational incentives for health providers practising in underserved and remote areas.^39^^,^^40^ In this regard, workforce supportive supervision, that is, a process of helping staff improve their own work performance based on the promotion of mentorship, problem-solving, and two-way communication, may serve to empower health care providers to continuously improve their performance.^41^ Supportive supervision can enhance local clinical practice, reduce demotivation, and mitigate attrition and professional emigration rates.^42^, ^43^, ^44^, ^45^, ^46^

We did not identify eligible papers to be included that addressed coronavirus disease 2019 (COVID-19) pandemic workforce shortages. We note that the COVID-19 pandemic has represented a major challenge for the availability and distribution of the workforce, and mitigation strategies have been put in place. An increase in the workload and telemedicine were two key strategies implemented to face the pandemic health demands.

Our research was developed to inform policy for the cancer workforce, as part of global cancer control efforts. The implementation of effective policies to orient investment in the workforce is one pillar of the 2017 WHO Health Assembly ‘*Cancer Resolution*’, and broadly, of the Sustainable Development Agenda, as outlined in the *WHO 2030 Global Strategy on Human Resources for Health.*^11^^,^^47^ We synthesized the body of the evidence intended to help operationalize the formulation of evidence-informed policies for the cancer workforce. Our work is the first of this type in the literature on oncology, based on our knowledge.

The exploration of economics and financing in the cancer workforce within low-resource settings is a crucial but overlooked aspect of global cancer treatment initiatives. This knowledge gap hinders progress, as limited financial resources in LICs and MICs pose challenges to providing fair and sustainable care. To address this barrier, it is necessary to consider the financial constraints when selecting interventions and planning for the long term. The existing focus on local research in current literature provides a strong basis for future financial modelling at a local level. Such modelling is essential for guiding intervention selection and setting future funding goals to achieve optimal outcomes within a given budget. To define which interventions are viable in resource-limited settings, future studies should incorporate financial models whenever possible, prioritizing cost-effectiveness as a key factor.

#### Strength and limits of our study

We restricted the scope of this study to the cancer workforce across the cancer continuum: this choice might have limited the evaluation of highly effective strategies that can have broader applicability but be implemented in a noncancer setting. We tried to limit the assumptions and extrapolations across disease areas, as cancer care is commonly multidisciplinary and complex, and sometimes issues and challenges faced in cancer control are unique, and better addressed with *ad hoc* cancer policies, albeit integrated across health care levels and disease programs.^48^^,^^49^ The data we collected may not capture most home-grown training programs and policies for strengthening these programs and building capacity, which may explain why most interventions are based on partnerships with HICs. Still, this is the first systematic review focusing on policy solutions and workforce interventions that scale up the cancer workforce. The use of AAAQ, SWOT, and the WHO labour market approach to analyse the evidence from the literature helped to characterize the strategies under multiple aspects, map the potential implications, and comprehensively embrace the body of the evidence.

In conclusion, our research provides a set of reference strategies for cancer workforce capacity building and scale up. Policies for the cancer workforce should pursue competency-based goals that are driven by quality indicators and oriented toward acceptability and culturally sensitive approaches. Solutions must be envisioned in the context of managerial solutions, including role delegation and *ad hoc* incentives to enforce health care in underserved areas, embedded in national, regional, and global health goals, with the intent to deliver health for all. Ultimately, tangible interventions on the cancer workforce should be measured as population health impact, with the aim of operationalizing comprehensive cancer control plans.
