# Supplementary material for: Policy strategies for capacity building and scale up of the workforce for comprehensive cancer care: a systematic review
Source: ESMO Open. 2024 Mar 19;9(4):102946. doi: 10.1016/j.esmoop.2024.102946 (PMC10966170; doi:10.1016/j.esmoop.2024.102946)

Supplementary material.

**Supplementary Table 1**. Synoptic table of the literature evidence extracted from the systematic review of the literature.

| **Year of intervention** | **Country of intervention** | **Committer** | **Country of the committer** | **Nature of commitment** | **Occupation(s) addressed** | **Type of study** | **Method of data collection** | **Title of the project** | **Delivery of intervention** | **Territorial setting** | **Health setting** | **Cancer continuum of care** | **Target a specific cancer type** | **Financial support specified** | **Reference** | **PMID/ DOI** |
| --- | --- | --- | --- | --- | --- | --- | --- | --- | --- | --- | --- | --- | --- | --- | --- | --- |
| from 2009 | US (North Carolina) | Duke University | US | academic | multidisciplinary team (occupations not specified) | observational | interview or questionnaire with providers | Duke Cancer Network platform for multidisciplinary tumor conferences | from remote | rural/ remote | Outpatient | treatment | yes, Lung cancer | no | *Stevenson MM, J Oncol Pract, 2013* | *PMID: 23942505* |
| from 2008 | US (California) | UC Davis Cancer Center | US (California) | academic | medical oncologist, surgical oncologist, pathologist, cancer nurse, radiation oncologist | observational | interview or questionnaire with providers | Virtual interactive multidisciplinary cancer tumor boards (VTBs) | from remote | rural/ remote | Outpatient | treatment | yes, breast cancer, yes, Lung cancer, GU cancer, | no | *Bold RJ, Commun Oncol, 2013* | *DOI: 10.12788/j.cmonc.0064* |
| 2014 - 2015 | Tanzania | Queen’s University (Kingston, Ontario Canada) | Canada | academic | cancer nurse, assistant medical officer | observational | record review |  | from remote | rural/ remote | Outpatient | early diagnosis/ screening | yes, cervical cancer | no | *Yeates KE, JGO, 2016* | *DOI: 10.1200/JGO.2015.001768* |
| 2012 - 2015 | UK (London) | NHS Foundation Trust | UK (London) | public funding | urology nurse practitione | interventional; pre-post without randomization | framework of data collection in a randomized trial |  | in- country with interventions on site | urban/ peri-urban | Outpatient | early diagnosis/ screening | prostate cancer | no | *Drudge-Coates L, Eur Urol Suppl, 2018* | *Eur Urol Suppl 2018; 17(2);e1871* |
| 2011– 2012 | UK |  | UK | public funding | non-medical genetic counsellor | observational | interview or questionnaire with patients |  | in- country with interventions on site | urban/ peri-urban | Outpatient | prevention | no | yes | *Benjamin C, Eur J Hum Genet, 2015* | *PMID: 25758997* |
| 2011 - 2013 | Pakistan | Health system, Pink Ribbon Pakistan and Aga Khan Health system, UICC | Pakistan | public funding | clinical oncologist, cancer nurse, radiologist | interventional, pre-post without randomization | mammographies performed | Mobile Mammography | in- country with interventions on site | rural/ remote | community setting | early diagnosis/ screening | yes, breast cancer | yes | *Laghari NA, Asia-Pacific Journal of Clinical Oncology, 2014* | *http://wcc-2014.p.asnevents.com.au/tracks/657/abstract/16728* |
| 2009 - 2011 | Australia |  | Australia | academic | medical oncologist | observational | record review | Townsville Teleoncology Model | from remote | rural/ remote | mixed in-outpatient | treatment, palliation of cancer | no | no | *Sabesan S, Aust. J Rural Health, 2014* | *doi: 10.1111/ajr.12101* |
| 2009 - 2011 | US | American Society of Clinical Oncology (ASCO) | US | NGO | Non-physician practitioners (NPP) | observational | interview or questionnaire with providers | Study of Collaborative Practice Arrangements (SCPA) | in- country with interventions on site | mixed | mixed in-outpatient | early diagnosis/ screening, treatment, palliation of cancer, supportive care (including psychoncology) | no | yes | *Towle EL, J Oncol Pract, 2011* | *PMID: 22211119* |
| 2008 - 2011 | US (North Carolina) | Duke University | US (Norh Carolina) | academic | pathologist, intended as geneticist | interventional; post-only with randomized control | interview or questionnaire with patients | Telegenetics | mixed in-site and from remote | rural/ remote | Outpatient | prevention | no | yes | *Buchanan AH, J Genet Couns, 2015* | *PMID: 25833335* |
| 2008 - 2009 | US (Washington, Alaska) | Native People for Cancer Control Telehealth Network (NPCCTN) | US | academic | Rural healthcare providers (not specified) | observational | interview or questionnaire with providers |  | from remote | rural/ remote | community setting | cancer education | no | no | *Doorenbos ZA, J Cancer Educ, 2011* | *doi:10.1007/s13187-011-0204-4* |
| 2008 - 2009 | India | JivDaya foundation | US | NGO | cancer nurse, 1 medical social worker, 1data manager, 1outreach worker | interventional; interrupted time- series/ quasi- experimental | record review |  | in- country with interventions on site | rural/ remote | hospital inpatient ward | treatment, palliation of cancer, supportive care (including psychoncology) | yes, pediatric cancer | yes | *Mehta P, Pediatric Blood and Cancer, 2013* | *System Id: 20438070* |
| 2007 - 2011 | Australia (North Queensland and the Gulf of Carpentaria) | Townsville Cancer Centre | Australia | public funding | nurse, local medical officer | interventional; post-only, without randomized control | interview or questionnaire with providers |  | from remote | rural/ remote | Outpatient | treatment | no | yes | *Mooi JK, Aust. J. Rural Health, 2012* | *PMID: 22998201* |
| 2007 - 2011 | US |  | US | private industry | breast surgery nurse practitioners | interventional; pre-post without randomization | record review |  | in- country with interventions on site | urban/ peri-urban | mixed in-outpatient | treatment | yes, breast cancer | yes | *Kanumuri P, Annals of Surgical Oncology, 2013* | *System Id: 20441698* |
| 2007 - 2009 | Guatemala | St. Jude, the University of Tennessee Health Science Center; the Medical School of Francisco Marroquín, University of Guatemala | US | academic | paediatric cancer nurse | interventional; pre-post without randomization | external monitoring committee | International Outreach Nursing Program | in- country with interventions on site | rural/ remote | mixed in-outpatient | treatment, palliation of cancer, supportive care (including psychoncology) | yes, pediatric cancer | yes | *Day SW, Pediatr Blood Cancer, 2013* | *doi:10.1002/pbc.24318* |
| 2006 - 2010 | Ghana |  | Norway | public funding, academic | pathologist | observational | record review |  | mixed in-site and from remote | urban/ peri-urban | Outpatient | early diagnosis/ screening, diagnosis | no | yes | *Stalsberg H, Cancer, Cancer, 2008* | *DOI 10.1002/cncr.23830* |
| 2005 - 2007 | Guyana | Association canadienne des chirurgiens généraux (ACCG). | US | public funding | surgical oncologist, cancer and non-cancer surgeon | observational | record review |  | in- country with interventions on site | urban/ peri-urban | mixed in-outpatient | treatment | no | yes | *Cameron BH, Can J Surg, 2010* | *PMID: 20100407* |
| 2004 - 2006 | Jordan | Hospital for Sick Children, Toronto | Canada | academic | paediatric oncologist, neuro-radiologist, neurosurgeon, radiation oncologist | observational | record review | The Jordanian-Canadian Telemedicine on Pediatric Neuro-Oncology | from remote | urban/ peri-urban | mixed in-outpatient | treatment, palliation of cancer, supportive care (including psychoncology) | yes, pediatric cancer, Brain and spinal paediatric tumours | no | *Qaddoumi I, Pediatr Blood Cancer, 2007* | *DOI 10.1002/pbc.21085* |
| 2004 - 2005 | UK (Scotland) |  | UK (Scotland) | academic | clinical oncologist, medical oncologist, surgical oncologist, pathologist, cancer nurse, radiologist | interventional; pre-post with randomized control | record review | TELEMAM | mixed in-site and from remote | rural/ remote | mixed in-outpatient | treatment | yes, breast cancer | no | *Kunklera IH, European Journal of Cancer , 2 0 0 7* | *doi:10.1016/j.ejca.2007.08.026* |
| 2004 - 2005 | UK | University of Edinburgh | UK | academic | clinical oncologist, medical oncologist, surgical oncologist, pathologist, cancer nurse, radiation oncologist | interventional; pre-post with randomized control | external monitoring committee | TELEMAM | from remote | rural/ remote | Outpatient | treatment, palliation of cancer, supportive care (including psychoncology) | yes, breast cancer | no | *Kunkler IH, Eur J Cancer, 2007* | *PMID: 17962011* |
| 2002 - 2004 | US |  | US | public funding | patient navigator | interventional; pre-post without randomization | record review | IMPAACT (Improving Patient Access and Adherence to Cancer Treatment) trial | in- country with interventions on site | mixed | Outpatient | treatment | yes, breast cancer, gynecologic cancers | yes | *Ell K, Cancer, 2009* | *PMID: 19551881* |
| 2000 - 2009 | Tanzania | Pathologists beyond borders (Associazione Patologi Oltre Frontiera) | Italy | NGO | pathologist | observational | record review |  | in- country with interventions on site | urban/ peri-urban | mixed in-outpatient | treatment, diagnosis | no | yes | *Tumino R, Infectious Agents and Cancer, 2017)* | *DOI 10.1186/s13027-017-0115-z* |
| 1998 - 2004 | Sudan | Comitato Collaborazione Medica (CCM) | Italy | academic, NGO | general and cancer surgeon | interventional; post-only with randomized control | record review | Operation Lifeline Sudan (OLS) | in- country with interventions on site | rural/ remote | mixed in-outpatient | treatment | no | yes | *Meo G, World J Surg, 2006* | *https://doi.org/10.1007/s00268-005-0093-y* |
| 1998 - 2000 | USA (California) | University of California at Los Angeles (UCLA). | USA | public funding | radiology technician, radiology nurse | interventional; pre-post with randomized control | interview or questionnaire with patients | health education plus on-site mobile mammography (Mobile Mammography) | in- country with interventions on site | urban/ peri-urban | community setting | early diagnosis/ screening | yes, breast cancer | no | *Naeim A, J Am Geriatr Soc, 2009.* | *DOI: 10.1111/j.1532-5415.2008.02105.x* |
| 2017 | US (Virginia) | urologic allied health professionals (AHP) | US (Virginia) |  | nurse practitione | interventional; post-only without control | NR |  | mixed in-site and from remote | urban/ peri-urban | Outpatient | diagnosis, follow-up | bladder cancer | no | *Lee H, Journal of Urology, 2017* | *https://www.jurology.com/article/S0022-5347(17)33369-4/pdf* |
| 2017 | India | Harvard's EdX platform, Harvard University | US | academic | pathologist | interventional; pre-post without randomization | interview or questionnaire with providers | Massive Open Online Course for Pap smear technology (MOOC) | from remote | rural/ remote | Outpatient | early diagnosis/ screening | yes, cervical cancer | yes | *Dewar R, Laboratory Investigation, 2017* | *System Id: 20441122* |
| 2016 | Rwanda | Dana-Farber Cancer Institute | US | academic | cancer nurse | interventional; pre-post without randomization | interview or questionnaire with providers | oncology nursing education and skill development | in- country with interventions on site | rural/ remote | mixed in-outpatient | treatment, palliation of cancer, supportive care (including psychoncology), survivorship, follow-up | no | yes | *Muhayimana C, Annals of Global Health, 2016* | *http://doi.org/10.1016/j.aogh.2016.04.324* |
| 2014 | US (Alabama) | University of Alabama, Birmingham, AL | US (Alabama) | academic | community health advisors | observational | interview or questionnaire with providers |  | in- country with interventions on site | urban/ peri-urban | Outpatient | palliation of cancer | no | no | *Kvale E, Journal of Pain and Symptom Management, 2014* | *DOI: https://doi.org/10.1016/j.jpainsymman.2013.12.157* |
| 2013 | Australia |  | Australia |  | radiation oncologist, radiation therapist | observational | record review | Better Access to Radiation Oncology Workforce Innovation grant | from remote | rural/ remote | Outpatient | treatment, palliation of cancer | no | no | *Enge M, Journal of Medical Imaging and Radiation Oncology, 2014* | *System Id: 20441638* |
| 2013 | Australia | SMICS, University of Monash | Australia | academic | health care professionals in supportive care of cancer patients | interventional;pre-post without randomization | interview or questionnaire with providers | online supportive care training program | from remote | mixed | Outpatient | palliation of cancer | no | no | *Brady L, Asia-Pacific Journal of Clinical Oncology, 2013* | *https://doi.org/10.1111/ajco.12148_4* |
| 2013 | Malawi | partnership between Kamuzu Central Hospital (KCH) Malawi, the University of Malawi-College of Medicine, the University of North Carolina in the United States, and Haukeland University Hospital, Norway |  | academic | surgical oncologist | observational | descriptive analysis | College of Surgeons of East, Central and Southern Africa (COSECSA) surgical trainig program | in- country with interventions on site | urban/ peri-urban | educational program | treatment | no | yes | *Qureshi JS, Surgery, 2013* | *PMID: 23063312* |
| 2012 | US | School of Medicine, University of Pittsburgh | US | academic | pathologist | observational | financial analysis of 5 years- projected cost savings |  | from remote | mixed | mixed in-outpatient | early diagnosis/ screening | yes, breast cancer, melanoma | no | *Ho J, J Pathol Inform, 2014* | *DOI: 10.4103/2153-3539.139714* |
| 2012 | Canada (Ontario) | Cancer Care Ontario (CCO), Ministry of Health and Long-Term Care (MOHLTC) | Canada | public funding | Radiation Oncologist, Medical Physicists, Radiation Therapists | interventional; interrupted time- series/ quasi- experimental | evaluation of facility and service delivery rate |  | in- country with interventions on site | mixed | Outpatient | treatment | no | yes | *Ang M, JCO, 2014* | *PMID: 28141244* |
| 2012 | Mexico | Centro Nacional de Equidad de Género y Salud Reproductiva and Dirección General de Programación y Presupuesto | Mexico | public funding | radiographer (radiologic technologist) | interventional; pre-post without randomization | external monitoring committee |  | in- country with interventions on site | urban/ peri-urban | Outpatient | early diagnosis/ screening | yes, breast cancer | yes | *Torres-Mejía G, BMC Cancer, 2015* | *DOI 10.1186/s12885-015-1399-2* |
| 2012 | Kenya | University of Toronto | Canada | academic | clinical oncologist, medical oncologist, surgical oncologist, pathologist, paediatric oncologist, radiation oncologist, ophthalmic clinical officers, nurses | observational | interview or questionnaire with providers | 2013 Kenyan National Retinoblastoma Strategy meeting | in- country with interventions on site | urban/ peri-urban | educational session (workshop) | training and education | retinoblastoma | yes | *Hill JA, PLOS one, 2015* | *DOI:10.1371/journal.pone.0129852* |
| 2012 | Italy | Azienda Provinciale per i Servizi Sanitari, Trento (Italy) | Italy | public funding | pathologist | interventional; pre-post without randomization | performance assesment | Computer-assisted Pap test for cervical cancer screening | in- country with interventions on site | urban/ peri-urban | Outpatient | early diagnosis/ screening | yes, cervical cancer | yes | *Della Palma P, Epidemiol Prev, 2012* | *23139174* |
| 2011 | US | Cancer Prevention and Research Institute of Texas (CPRIT) | US | academic | cancer nurse | observational | interview or questionnaire with providers | Professional Education for Prevention and Early Detection (PEPED) program | in- country with interventions on site | mixed | community setting, Outpatient | early diagnosis/ screening | no | yes | *Dallred CV, J Canc Educ, 2012* | *DOI 10.1007/s13187-012-0326-3* |
| 2010 | US (Indiana) | PA Labs in Indianapolis | US (Indiana) | public funding | pathologist | interventional; pre-post without randomization | interview or questionnaire with providers |  | in- country with interventions on site | urban/ peri-urban | laboratory | early diagnosis/ screening | yes, cervical cancer | yes | *Elsheikh TM, Cancer Cytopathology, 2010* | *DOI: 10.1002/cncy.20065* |
| 2009 | US (Florida) |  | US | public funding, private industry, academic | primary care physicians, nurses, social workers, and office staff | interventional; pre-post without randomization | interview or questionnaire with providers | 3 University of Florida—Pain and Palliative Care Competency Training for Non-Oncology Health Professionals Working in Rural Settings | in- country with interventions on site | rural/ remote | community setting | palliation of cancer | no | yes | *Cox KA, J Canc Educ, 2012* | *DOI 10.1007/s13187-012-0354-z* |
| 2009 | US (Virginia) | C-Change | US | public funding, private industry, academic | 4y medical students | interventional; pre-post without randomization | interview or questionnaire with providers | Virginia Commonwealth University—Pediatric Pain Management: The Development of an Online Competency Modul | in- country with interventions on site | urban/ peri-urban | mixed in-outpatient | palliation of cancer | yes, pediatric cancer | yes | *Cox KA, J Canc Educ, 2012* | *DOI 10.1007/s13187-012-0354-z* |
| 2009 | US (Iowa) |  | US (Iowa) | public funding, private industry, academic | medical assistants, nurses | interventional; pre-post without randomization | interview or questionnaire with providers | C-Change: Iowa Cancer Coalition—End of Life Communication and Collaboration | in- country with interventions on site | rural/ remote | hospital inpatient ward | palliation of cancer | no | yes | *Cox KA, J Canc Educ, 2012* | *DOI 10.1007/s13187-012-0354-z* |
| 2009 | US | C-change | US | public funding, private industry, academic | ▪ Native health workers, cancer survivors, and caregivers from the five tribes | interventional; pre-post without randomization | interview or questionnaire with providers | 5 South Puget Intertribal Planning Agency—Addressing Culture-Specific Pain Management: Creating a Common Ground Between Community Members and Caregivers to Address Native American Cancer Pain and Palliative Care | in- country with interventions on site | rural/ remote | community setting | palliation of cancer | no | yes | *Cox KA, J Canc Educ, 2012* | *DOI 10.1007/s13187-012-0354-z* |
| 2006 | Nigeria | University of Chicago | US | academic | pathologist | interventional; pre-post without randomization | record review | Web-based training for quality improvement in IHC for breast cancer | from remote | urban/ peri-urban | mixed in-outpatient | diagnostic | yes, breast cancer | no | *Oluwasola AO, Annals of Diagnostic Pathology, 2013* | *. http://dx.doi.org/10.1016/j.anndiagpath.2013.07.003* |
| 2000 | Chile, Columbia, Costa Rica, Dominican Republic, Ecuador, El Salvador, Guatemala, Honduras, Mexico, Panama, Peru, andVenezuela, Morocco | St Jude Hospital | US | academic | cancer nurse | interventional; pre-post without randomization | interview or questionnaire with providers | Teach the Teachers Program for Training Nurses in Pediatric Oncology | in- country with interventions on site | mixed | mixed in-outpatient | treatment, palliation of cancer, supportive care (including psychoncology) | no | yes | *Wilimas JA, Med Pediatr Oncol, 2003* | *DOI 10.1002/mpo.10242* |
| NR | England |  | England | academic | cancer nurse | interventional; pre-post with randomized control | interview or questionnaire with patients |  | in- country with interventions on site | urban/ peri-urban | Outpatient | supportive care (including psychoncology) | yes, Lung cancer, mestohelioma | no | *Moore S, European Journal ofOncology Nursing, 2004* | *PMID: 12433764* |
| NR | India (Bengal) |  | India (Bengal) | public funding | palliative care provider | observational | NR |  | from remote | rural/ remote | home- based care | supportive care (including psychoncology) | no | no | *Manna A, The Journl of pain, 2016* | *https://doi.org/10.1016/j.jpain.2016.01.120* |

**Supplementary figure 1. Market labour approach in the evaluation and policymaking for the health workforce according to the WHO 2030 Strategy.**


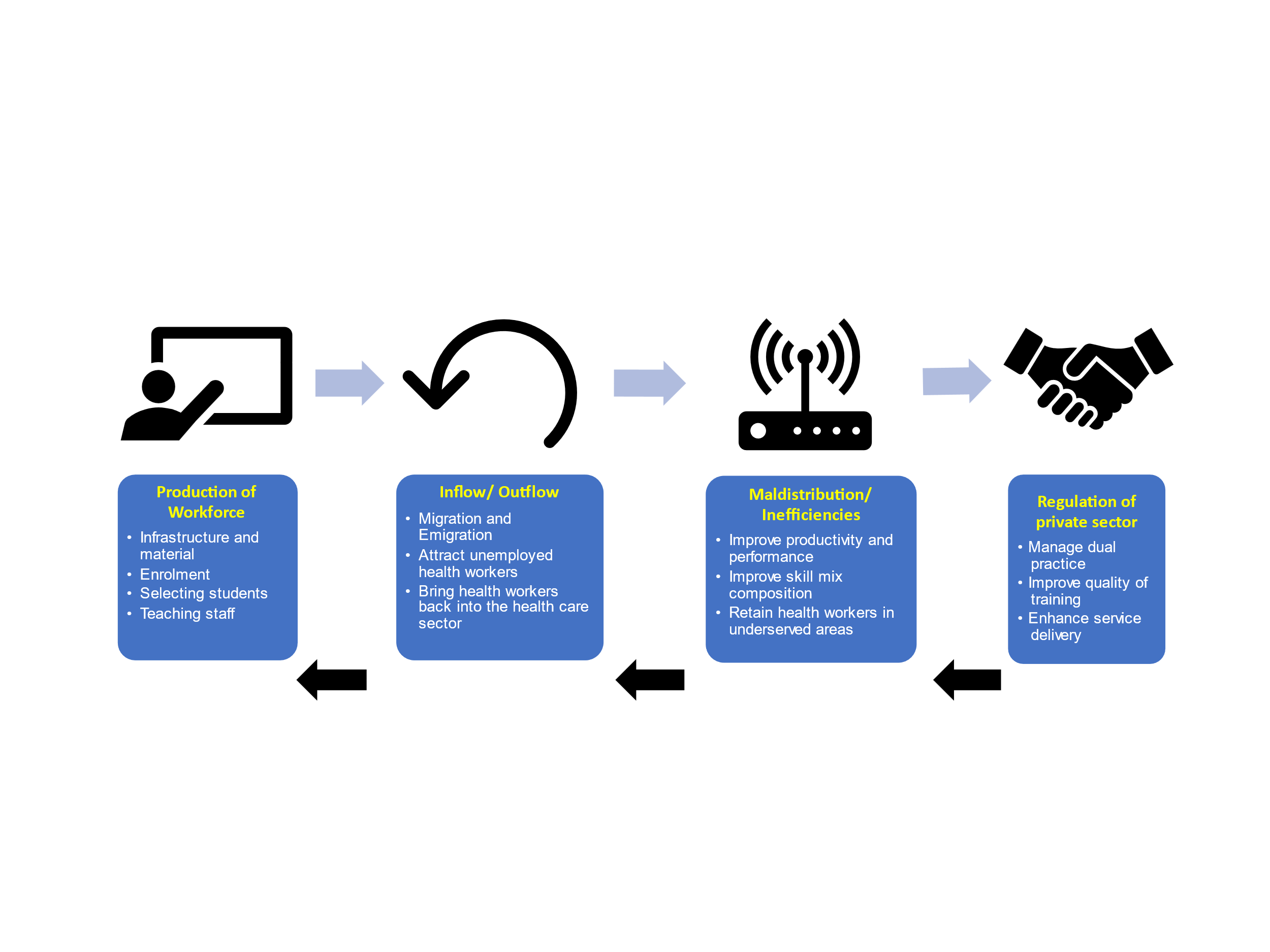


**Supplementary figure 2**. Number of providers and type of providers addressed by the strategies extracted from the systematic review of the literature.

1.
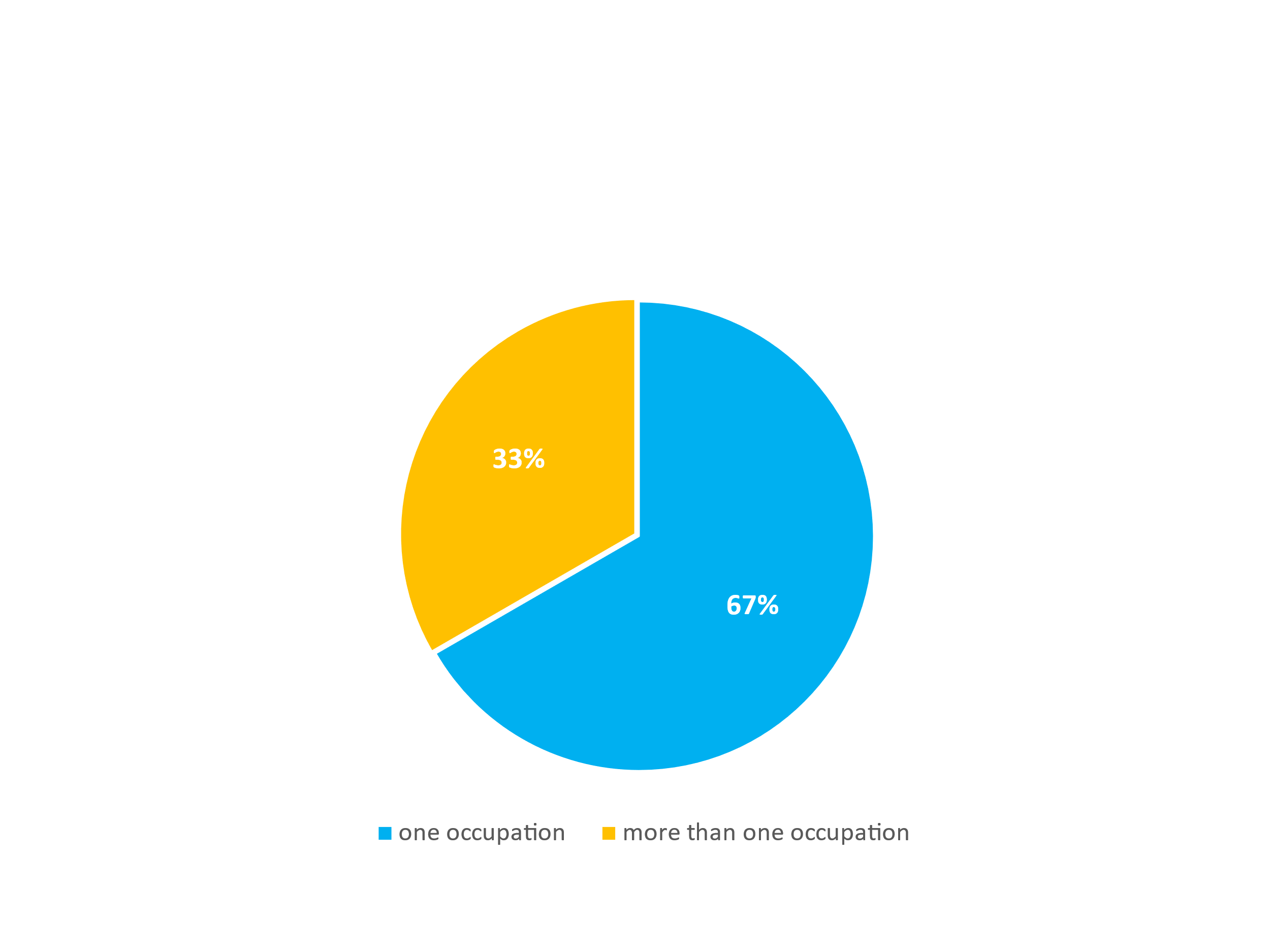
 B)
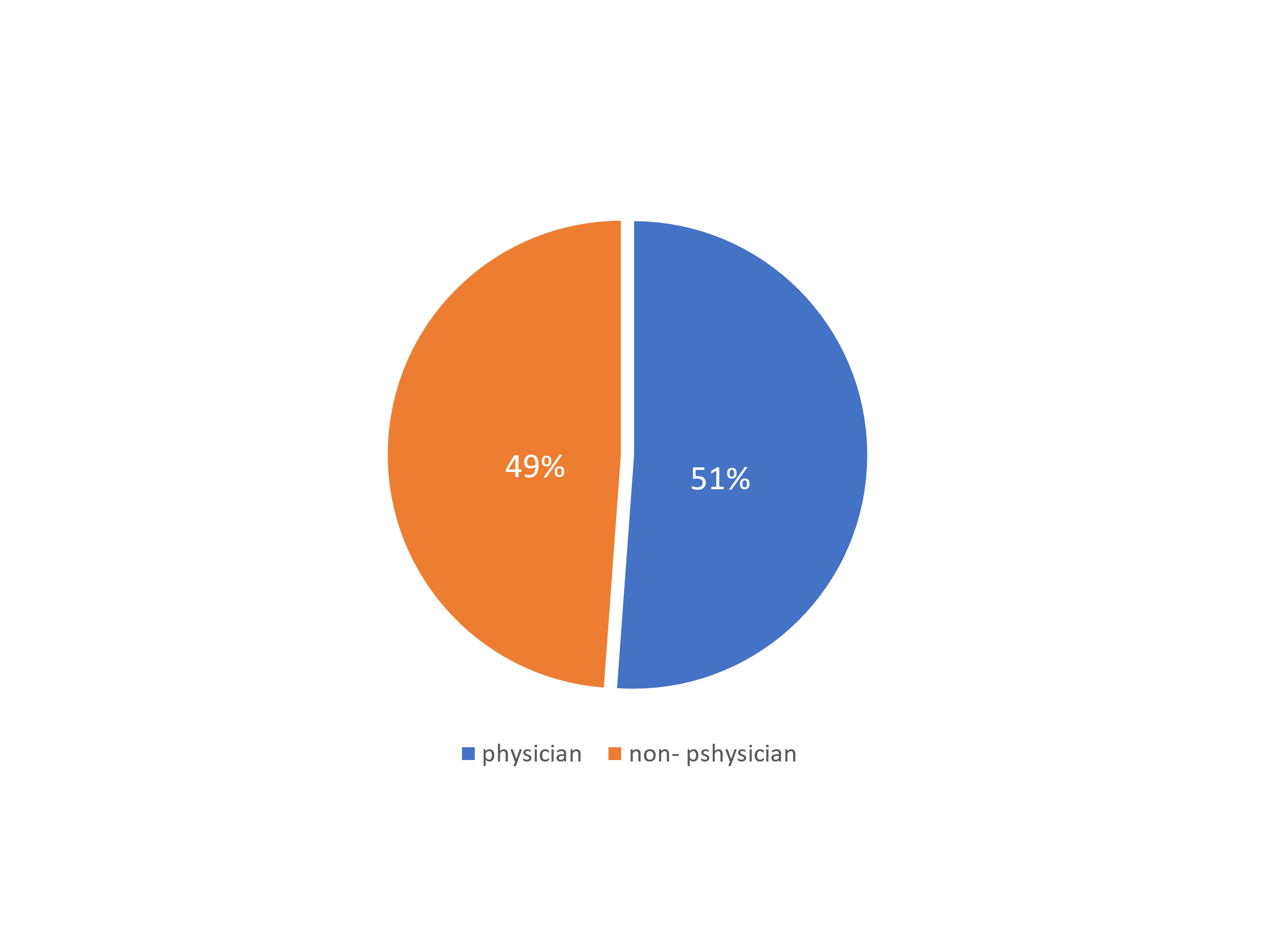


**Supplementary figure 3. Funding source (A) and type of institutions in charge of the development (B) of the workforce strategies identified in the systematic review of the literature.**

**A)**
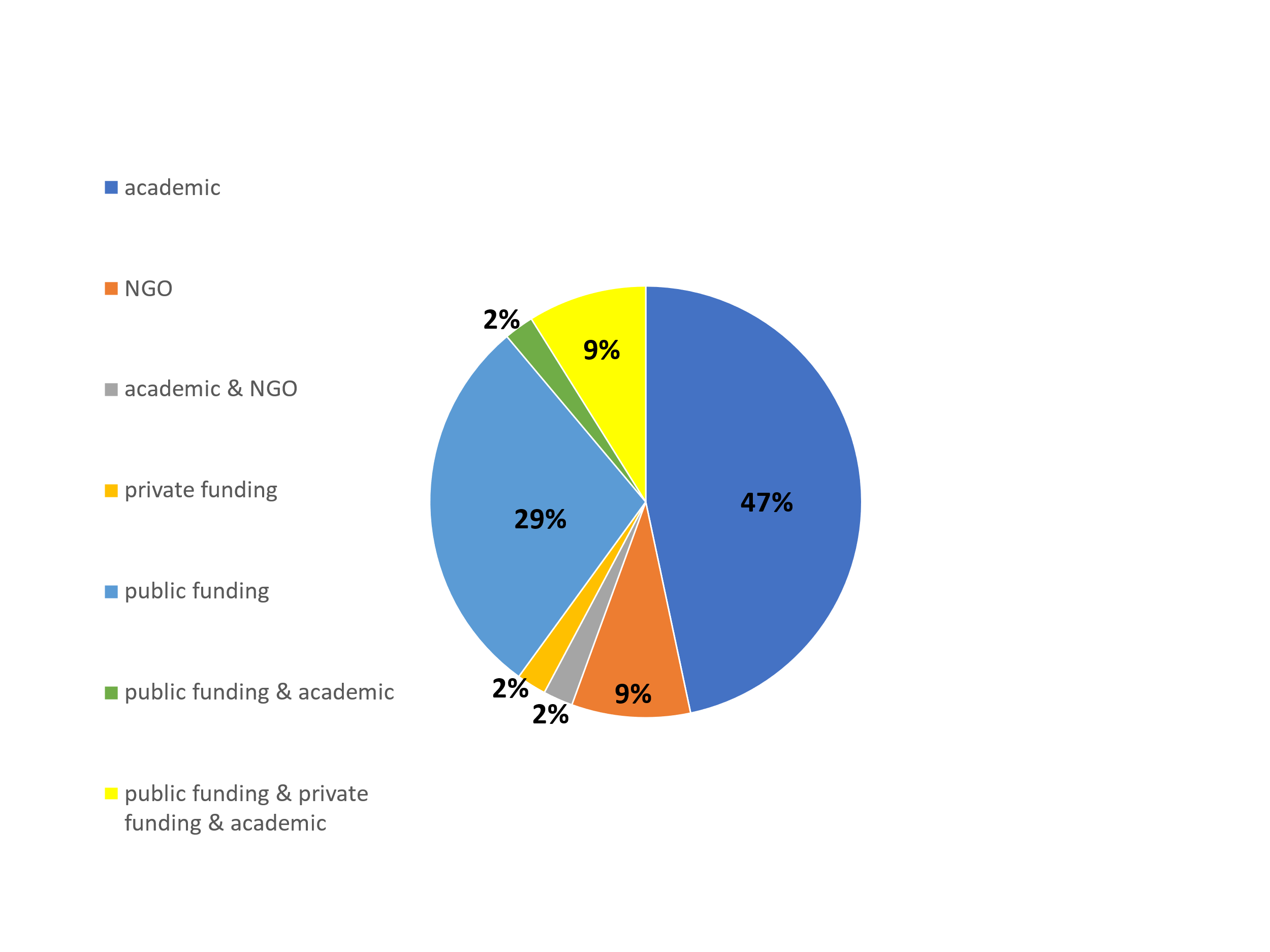
 **B)**
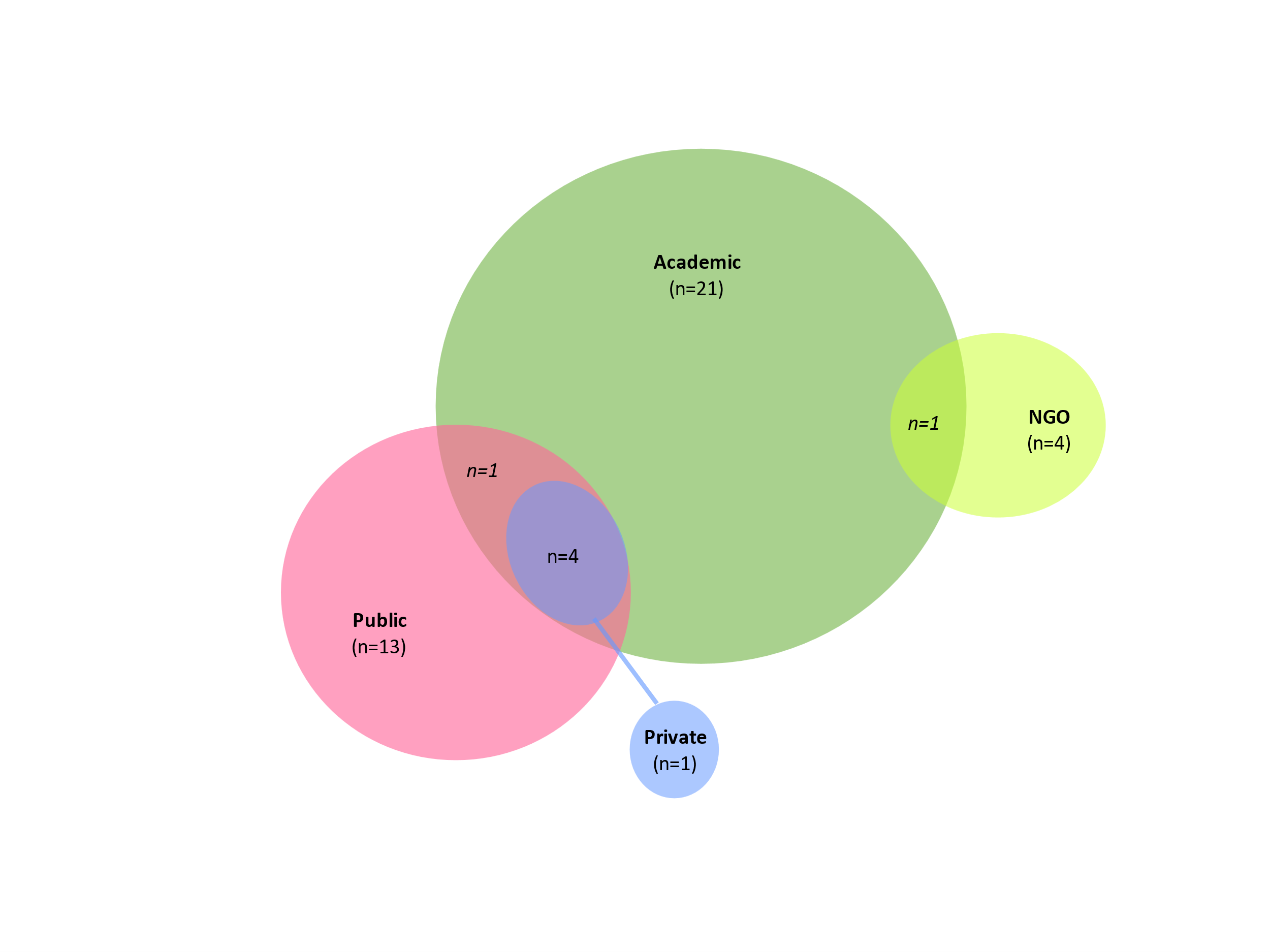


**Supplementary figure 4**. Type of delivery (A: on-site vs from remote) and setting of care (B: rural vs urban) where the workforce interventions where implemented.


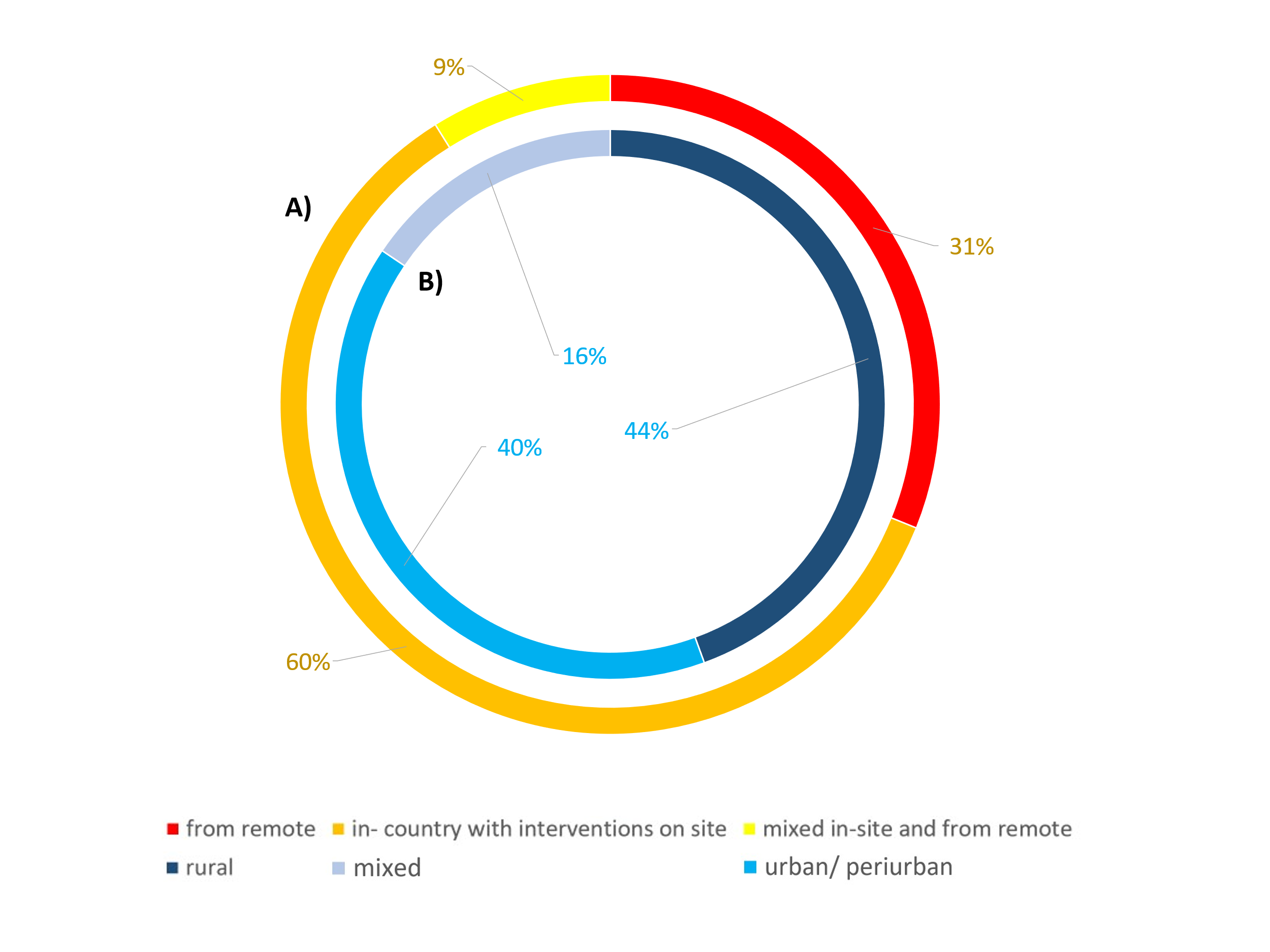


**Supplementary figure 5**. Methodology to evaluate the impact of the workforce strategies identified in the systematic review.


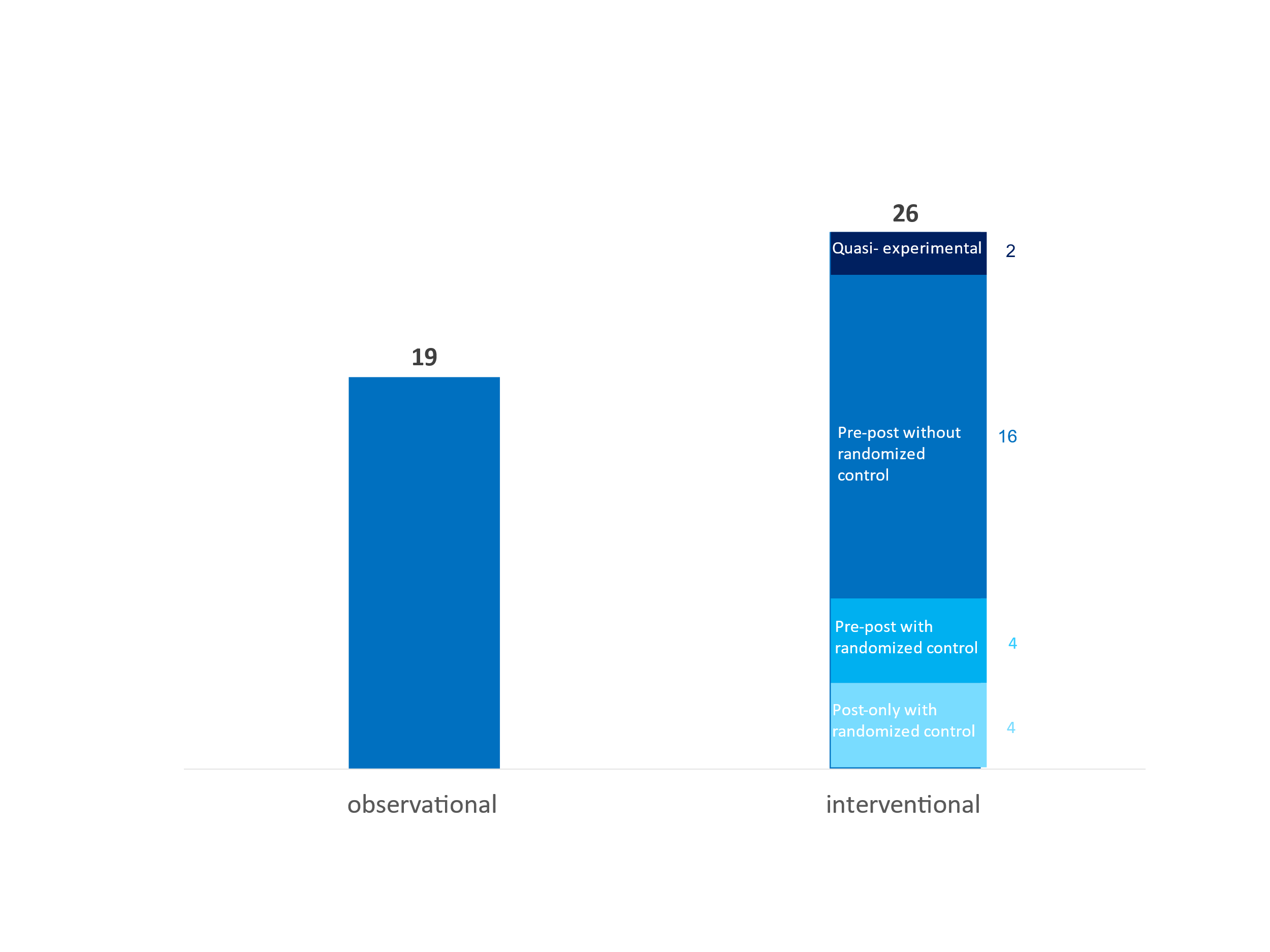


**Supplementary figure 6**. Impact of the workforce interventions, classified according to the WHO 2030 Strategy on five standardized scenarios of workforce capacity-building and scale-up. *Note that some studies address more than one sub-level from a level*.


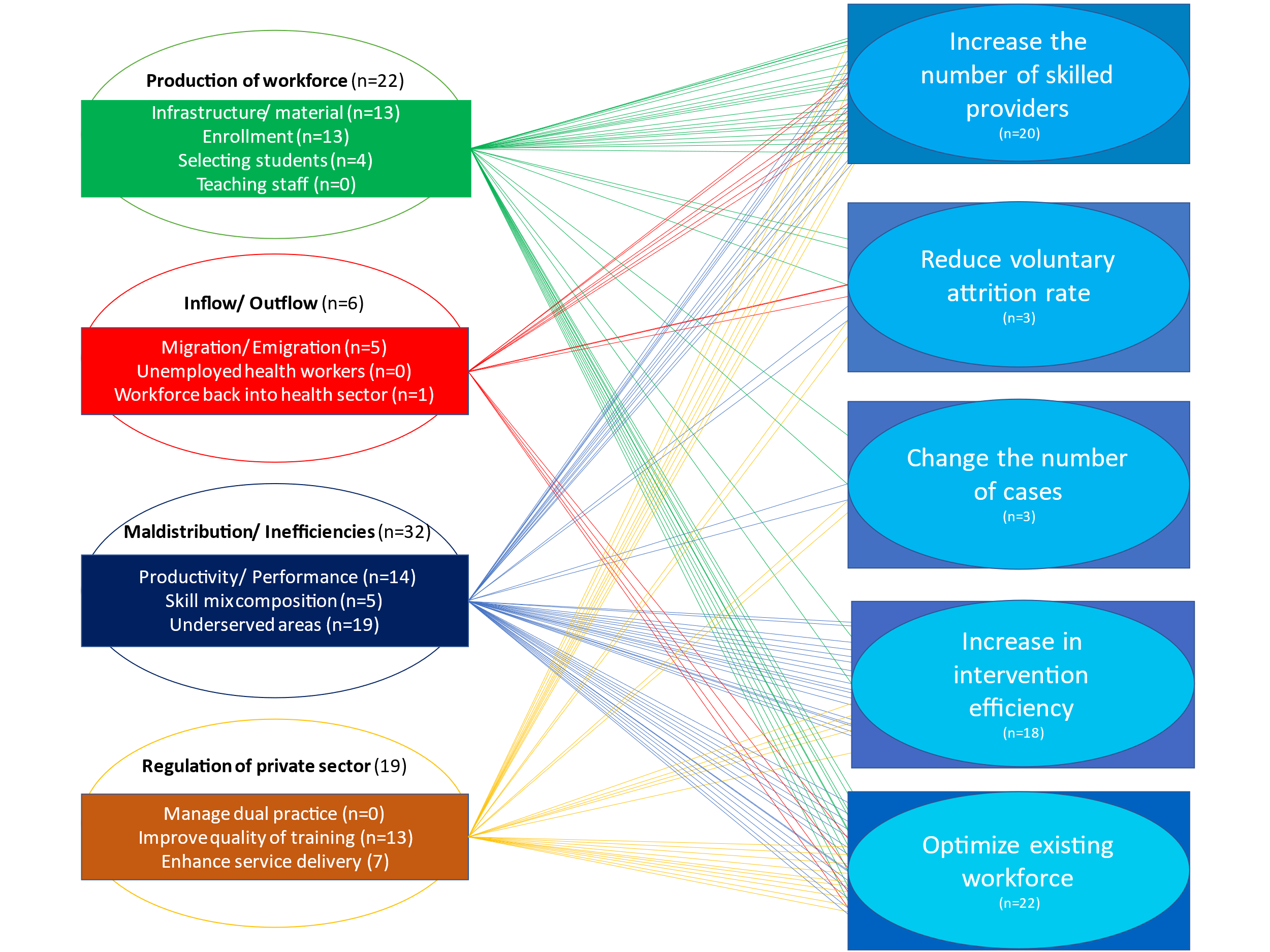

Supplement: Supplementary data [file mmc1.docx]
